# A comparative study of human glomerular basement membrane thickness using direct measurement and orthogonal intercept methods

**DOI:** 10.1186/s12882-021-02634-1

**Published:** 2022-01-10

**Authors:** Débora Leal Viana, Dona Jeanne Alladagbin, Washington L. C. dos-Santos, Claudio Pereira Figueira

**Affiliations:** 1 Oswaldo Cruz Foundation, Gonçalo Moniz Institute, Rua Waldemar Falcão 121, Candeal, Salvador, BA CEP 40.295-710 Brazil; 2 Faculty of Medicine, Federal University of Bahia, Salvador, BA Brazil

## Abstract

**Introduction:**

Here we report estimates of glomerular basement membrane (GBM) thickness in the Brazilian population performed using direct (DM) and orthogonal interception methods (OIM), and comment on potential sources of variation among estimates made by different laboratories.

**Methodology:**

A total of 38 patients, ranging from 3 to 78 years of age, 26 (68%) males and 12 (32%) females, were submitted to kidney biopsy procedures for renal disease diagnosis. Glomeruli were diagnosed with minor histological changes by conventional, immunofluorescence and electron microscopy. GBM thickness was estimated using both DM and OIM methods.

**Results:**

Estimates of GBM thickness obtained using DM were higher than those obtained by OIM. However, the application of a correction for non-perpendicular membrane sectioning to DM estimates yielded similar results to those obtained under OIM. The estimated GMB thickness using DM after correction was 289 + 44 nm, versus 287 + 48 nm by OIM. No statistically significant differences were detected in GMB thickness, nor with respect to patient age or sex.

**Conclusions:**

GBM thickness in the studied Brazilian population measured approximately 290 nm. The application of criteria for estimating the shortest distance between the endothelial and podocyte cell membranes with correction for non-perpendicular membrane sectioning can increase the accuracy of GBM thickness estimates using DM and OIM.

**Supplementary Information:**

The online version contains supplementary material available at 10.1186/s12882-021-02634-1.

## Introduction

Estimating glomerular basement membrane (GBM) thickness using transmission electron microscopy images is essential for the diagnosis of hereditary nephropathies (e.g. thin basement membrane disease and the early stages of Alport syndrome) and may also provide insight into the progression of diabetic nephropathy.

Some factors can interfere with the estimation of GBM thickness. Differences in composition between fixative solutions, as well as in embedding and polymerization processing, can introduce variability into estimated values [1, 2]. Therefore, it is recommended that every pathology laboratory perform its own calibration process. Additionally, the methodology used for estimating GBM thickness may constitute an additional source of variation among laboratories.

Two measuring systems are commonly used to estimate the thickness of GBMs: the direct measurement (DM) and orthogonal intersection (OIM) methods. DM is performed by making at least 16 estimates, 3 μm apart, in cross-sections of the GBM [3–5]. OIM involves performing measurements at all orthogonal intercepts along the subendothelial aspect of the GBM using a grid laid over the image in at least two glomeruli [2, 6, 7]. While DM is less time-consuming and easier to perform than OIM, the results obtained can be highly variable [2]. The application of corrections for non-perpendicular membrane sectioning are consistently mentioned in papers reporting estimated GBM thickness.

Nevertheless, reported GBM thickness varies across different studies, values lower than 300 or higher than 400 can be arbitrarily considered as abnormal [3]. Some authors have reported lower GBM thickness values in women and children than in male adults and little is known regarding the influence of ethnicity on GBM thickness [2, 8–10].

The present study, which aimed to contribute to the definition of more robust reference range values regarding GBM thickness, constitutes an initial attempt to determine normal GBM width in a highly admixed Brazilian population. The results obtained using a very simple and straightforward version of the DM technique were compared to those obtained using the OIM method. We call attention to the criteria used for estimating GBM thickness and the need to apply recommended corrections for non-perpendicular membrane sectioning. This study was conducted in the municipality of Salvador, Bahia-Brazil, whose population is characterized by predominantly Portuguese and African ancestry.

## Material and methods

### Patients and biopsies

The present cross-sectional study considered all cases of kidney biopsy submitted to diagnostic procedures at the Gonçalo Moniz Institute, Oswaldo Cruz Foundation (IGM-FIOCRUZ), located in Salvador, Bahia-Brazil. All cases were referred between 1998 and 2018 from three public nephrology services: the Roberto Santos General Hospital (HRS), Ana Nery Hospital (HAN), Santo Antonio Hospital (HSA) and Bahia State Children’s Hospital (HEC). Biopsied fragments were divided into three different samples for study by optical, transmission electron and immunofluorescence microscopy.

### Inclusion and exclusion criteria

Inclusion: The inclusion criteria adopted were all kidney biopsies without alterations on optic microscopy histological analysis.

Exclusion: To exclude patient with incipient chronic renal diseases, the following exclusion criteria were applied: (1) the presence of hematuria, defined as more > 3 red blood cells per high-power field (hpf) on urine sediment analysis; (2) a previous diagnosis of diabetes mellitus, laboratory test results revealing blood glucose concentration above 100 mg/dL, a previous history of high glucose blood concentration, or the use of glycemic control medication; (3) biopsy samples determined not representative of glomeruli on electron microscopic study, or those with processing artifacts that compromised the analysis of renal structures.

The study was conducted in accordance with the principles of the resolutions 466/12 of national health council of Brazil. The study protocol was approved by the local bioethics committee.

### Biopsy processing

Biopsied kidney fragments were divided into three sections and processed in accordance with one of the following protocols: (1) Fixation in formalin/acetic acid/alcohol, paraffin embedding, sectioning measuring 2–3 μm and staining with hematoxylin and eosin, Shiff’s periodic acid, Silver stein, Azan trichrome and Picrosirius red; (2) Fragments were embedded in freezing mounting media (TissueTek Oct), frozen, and cryostat sectioning was performed for direct immunofluorescence examination to detect IgA, IgG, IgM, Kappa and Lambda chains of immunoglobulins, as well as C1q, C3 and fibrinogen deposits; (3) Fragments were fixed in 2% glutaraldehyde in 0.1 M sodium cacodylate, post-fixed in 1% osmium tetroxide containing 0.8% iron thiocyanate and calcium chloride 5 mM – 0.1 M Sodium cacodylate Buffer, then embedded in PolyBed 812® medium and polymerized for 72 h at 60 °*C. ultra*-thin, approximately 70 nm thick, sections were obtained for examination under a Jeol 1230 electron microscope at 80 Kv. Digital images (10,000 × magnification) were used to estimate GBM thickness.

### Clinical and laboratory variables

Clinical and laboratory data were obtained from the biopsy request forms, the patient’s clinical records and pathologist reports to determine the applicability of exclusion criteria. The following additional data were obtained: age, sex, diagnosis of diabetes mellitus, use of glycemic control drugs, clinical diagnosis, histopathological diagnosis, presence of endocapillary, mesangial or extracapillary hypercellurity, interstitial inflammation, tubular casts, tubular atrophy, interstitial fibrosis, presence of immune complexes.

Patients presenting hematuria, diagnosis of diabetes mellitus or in use of glycemic control drugs, presenting glomerular hypercellularity or glomerular sclerosis were excluded from the study.

Table 1 lists the clinical characteristics of the included patients. The presence of nephrotic syndrome, characterized by proteinuria > 3.5 g/24 h, and acute kidney injury was defined according to the KDIGO definition.

**Table 1:** General characteristics of patients undergoing renal biopsies with normal glomeruli

PARAMETER	ESTIMATED VALUE	
N	38	(100%)
Sex		
male	**26**	**(68%)**
feminine	**12**	**(32%)**
Age (mean)	21.08	± (15.18)
Age ranges:		
0–10	10	(26%)
11–20	9	(24%)
21–30	10	(26%)
31–40	7	(18%)
41–50	0	(0%)
51–60	0	(0%)
> 60	2	(5%)
Clinical presentation:		
Nephrotic syndrome	31	(81.6%)
Non-nephrotic proteinuria	5	(13.2%)
Acute kidney injury	2	(5.2%)
Laboratory tests (median [q1-q3]):		
24 h proteinuria		3600 (3192–5490)
Serum creatinine		0.9 (0.7–1.4)
Histological diagnosis:		
Minimal Change Disease		38 (100%)

### Measurement of glomerular basement membrane thickness

Two methods, DM and OIM, were used to estimate GBM thickness using FIJI software [11].

**Fig. 1:**
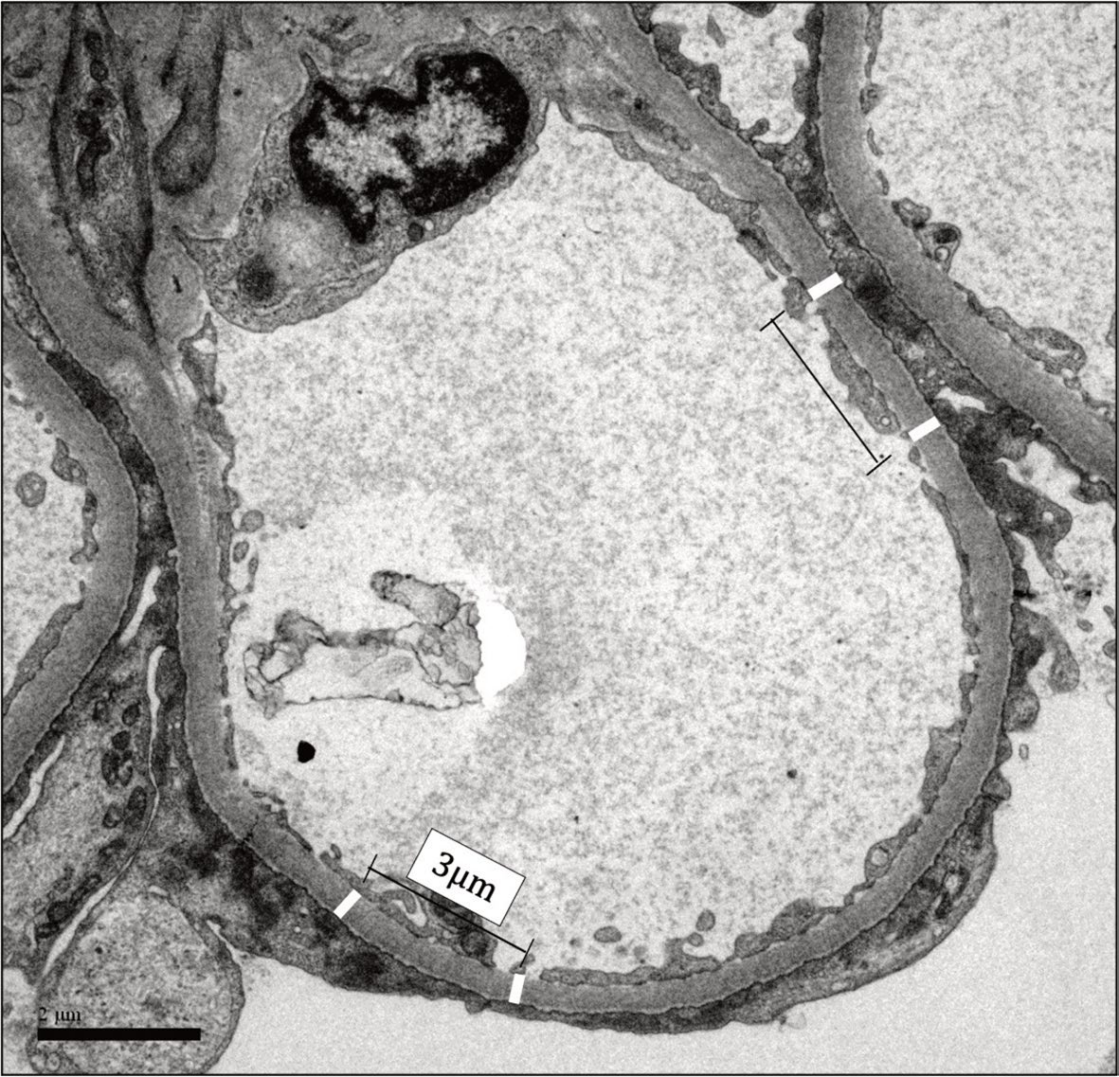
Electron micrograph of the glomerular loop showing Direct Method measurements of GBM thickness. Rectangles indicate points at which GBM thickness was measured. The distance between each point was 3 μm. The oblique angle in the loop was not measured

Direct measurement method: in glomerular endothelial cells, the distance was measured between the basal aspect of the endothelial membrane and the basal aspect of the podocyte membrane that touches the GBM. Measurements were made along uniform cross-sections of the glomerular capillary wall. Four images from non-overlapping areas were obtained at 10,000x magnification from each glomerulus under study. Each GBM thickness measurement was performed at least 3 μm apart from another. Three to five measurements were performed in each image, totaling 16 measurements in each set of 4 images (see Fig. 1). The thickness of each patient’s GBM was expressed as the arithmetic mean of the measurements [2–4].

**Fig. 2:**
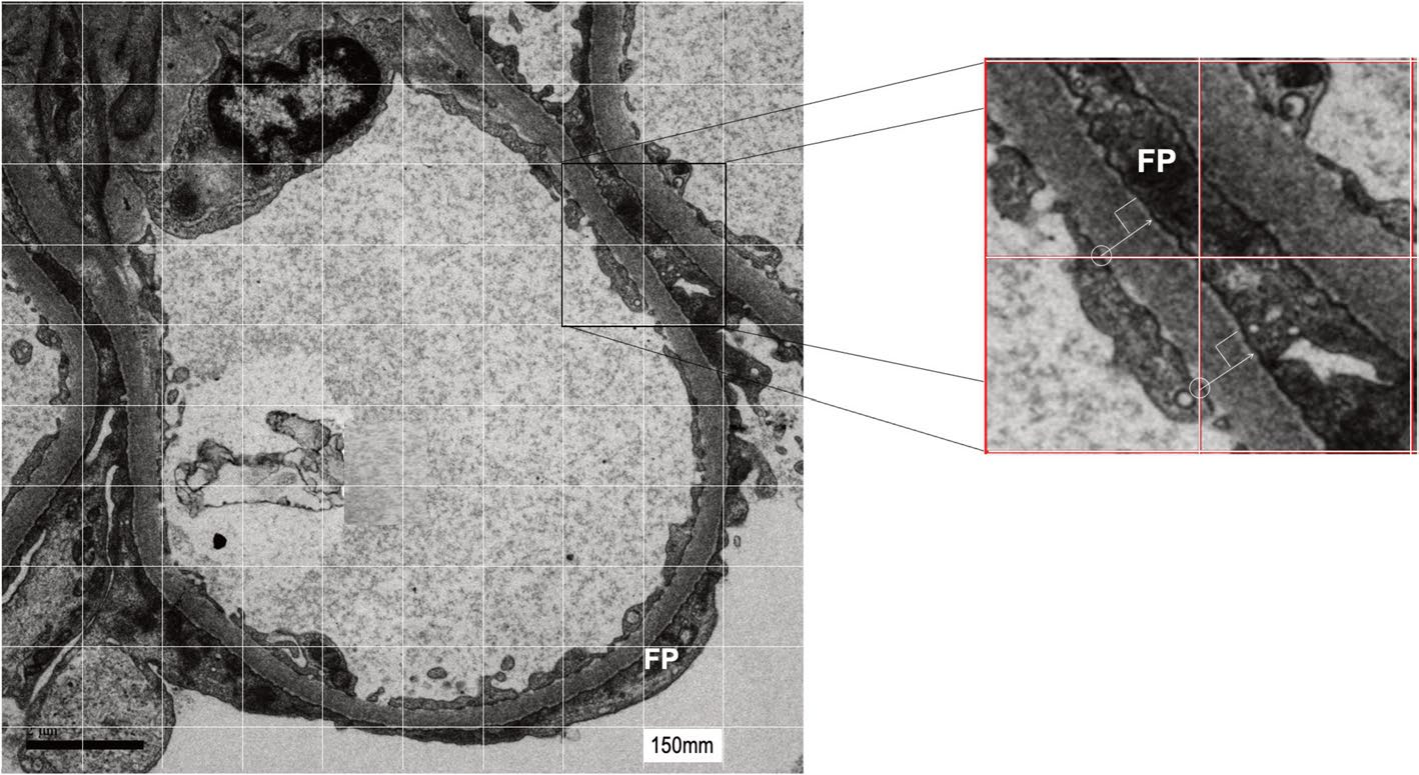
Electron micrograph of the glomerular loop showing Orthogonal Intercept Method measurements of GBM thickness. A square mesh grid (150x150mm) was laid over the micrograph image. All intercept points between the grid and the subendothelial aspect of the GBM were used reference measurements. Measurements were performed by tracing a perpendicular line (detail) from the subendothelial intercept point (circled) to the edge of the membrane along the foot process (FP)

Orthogonal interception method: Ten images from non-overlapping areas were obtained at 10,000x magnification only from patients who found two glomeruli. A 150 × 150 mm square mesh was placed over glomeruli images at 10,000x magnification [5–9]. All intercept points between the grid and the subendothelial aspect of the GBM were used for measurement references. Estimates were made at right angles across the GBM, as shown in Fig. 2. GBM thickness was expressed as the harmonic mean of the obtained measurements.

Corrections for non-perpendicular membrane sectioning were performed using one of the following formulas: T = 8/(3*π) (L_harmonic_), or T = (π/4)(L_O_), where T = estimated thickness, L_0_ = observed/measured thickness mean, and L_harmonic_, (the harmonic mean) represents the reciprocal of the mean of the reciprocals of L_0,_ the observed/measured thicknesses [7]. All the measurement was made by a Biomedical Sciences technician under supervision of a Pathologist.

Expression and analysis of results: All data were analyzed using Wizard version 1.9.42 (267) software. Results are presented as means or medians with accompanying dispersion estimates. The distribution of the variables was tested using D’Agostino’s K^2^ test. Correlations between DM and OIM estimates were investigated using the Pearson’s correlation coefficient test. Significance among the observed differences between two groups was tested using the Student’s t-test. Comparisons involving more than two groups were made using Tukey’s test. The critical level of significance was established as *p* < 0.05.

## Results

A total of 1621 renal biopsies were analyzed at IGM-FIOCRUZ during the study period. Of the 146 biopsies that presented minimal glomerular changes, 61 were obtained from patients with hematuria and four were from patients with clinical or laboratory signs of diabetes mellitus. In 43 patient biopsies, no samples were available for electron microscopic study, or the sample did not contain a glomerulus. In all, 38 patient biopsies were included in the study. The main clinical characteristics of these patients are described in Table 1.

Most patients were male (68%), with age varying between 3 and 78 years. No samples were obtained from patients aged between 41 60 years. Table 1 shows the distribution of patients by age range. Nephrotic syndrome was the main clinical presentation.

### Glomerular basement membrane estimates

Direct method: 16 measurements were performed in each glomerulus in each of the 38 patients, with a median of 32 [16–48] estimates per patient (Table 2). Overall mean DM GBM thickness was 369 ± 56 nm. After correcting for non-perpendicular membrane sectioning, the corrected DM GBM thickness value was 289 ± 44 nm. No statistical differences were observed among patients with respect to age range or sex (data not shown).

**Table 2:** Distribution of GBM thickness for age range using direct (DM), DM corrected and orthogonal intercept (OIM) methods and number of patients and measurements realized

AGE RANGES	DIRECT METHOD							ORTHOGONAL INTERSEPTION METHOD				
	PATIENTS	ESTIMATES ^a^		RESULTS^b^		RESULTS CORRECTED^b^		PATIENTS	ESTIMATES ^a^		RESULTS^b^	
0–10	10	32	[16–32]	356	± 57	279	± 46	7	177	[88–226]	263	± 55
11–20	9	32	[16–48]	370	± 55	290	± 45	6	164	[104–195	306	± 44
21–30	10	24	[16–32]	378	± 51	297	± 46	5	165	[70–210]	288	± 43
31–40	7	32	[16–48]	389	± 53	305	± 42	4	197	[186–210]	289	± 39
> 60	2	32	[16–48]	314	± 27	244	± 30	1	**256**		**291**	**± 58**
SUMMARY	38	32	[16–48]	369	± 56	289	± 44	23	165	[160–197]	287	± 48

^a^ Median [Q1 – Q3]. ^b^ Mean ± sd

Orthogonal interception method: 23 cases presented at least two glomeruli available for analysis. In all, 49 glomeruli were analyzed with a median of 165 [160–197] measurements performed per patient. After correcting for non-perpendicular membrane sectioning, mean OIM GBM was 287 ± 48 nm. No statistical differences were observed among the patients with respect to age or sex (data not shown).

### Comparisons between OIM and DM estimates

Comparisons of estimated GBM thickness by OIM and DM were only possible in 23 patients whose biopsied samples contained at least two glomeruli available for analysis. Strong correlation was observed between the estimates obtained using both methods [Pearson’s correlation (DM vs. OIM) r = 0.712, *P* < 0.0001]. mEstimates obtained by DM were found to be markedly higher than OIM values, with a corresponding ratio of 1.3 ± 0.2 using uncorrected DM estimates and 1.0 ± 0.1 according to corrected DM estimates (Table 3). The coefficient of variation was similar for OIM (16%) and DM (12%).

**Table 3:** Comparison between direct method (DM) and orthogonal interception method

PATIENT	SEX	MEASURATION METHOD			DM/OIM RATIO	
		DIRECT		ORTHOGONAL		
		NEAT	CORRECTED	INTERCEPT	NEAT	CORRECTED
A1	**M**	348.1	273.4	216.2	1.6	1.3
A2	**M**	402.5	316.1	259.7	1.5	1.2
A3	**M**	375.4	294.8	225.4	1.7	1.3
A4	**F**	385.1	302.4	275.0	1.4	1.1
A5	**M**	298.7	234.6	210.3	1.4	1.1
A6	**M**	452.5	355.4	368.9	1.2	1.0
A7	**F**	368.3	289.3	287.3	1.3	1.0
B1	**M**	414.9	325.9	345.9	1.2	0.9
B2	**M**	420.4	330.1	290.6	1.4	1.1
B3	**M**	384.8	302.2	295.2	1.3	1.0
B4	**M**	326.5	256.4	235.6	1.4	1.1
B5	**F**	464.9	365.2	357.4	1.3	1.0
B6	**M**	367.8	288.9	314.0	1.2	0.9
C1	**F**	395.5	310.6	284.0	1.4	1.1
C2	**F**	327.0	256.8	263.7	1.2	1.0
C3	**F**	321.5	252.5	234.0	1.4	1.1
C4	**M**	440.0	345.6	342.8	1.3	1.0
C5	**M**	350.3	275.1	317.8	1.1	0.9
D1	**M**	402.2	315.9	333.7	1.2	0.9
D2	**F**	351.9	276.4	277.4	1.3	1.0
D3	**M**	364.5	286.3	291.1	1.3	1.0
D4	**F**	308.0	241.9	253.1	1.2	1.0
**E1**	**M**	**284.4**	**223.4**	**291.3**	**1.0**	**0.8**
**MED ± SD**		**372.0 ± 48.6**	**292.1 ± 38.2**	**285.7 ± 45.2**	**1.3 ± 0.2**	**1.0 ± 0.1**

*M* Male; *F* Female; A to D = Age ranges: A = 0 to 10; B = 11 to 20; C = 21 to 30; D = 31 or more. DMc = Direct Method with correction. OIM = Orthogonal Interception Method. Pearson correlation (DMc x OIM) r = 0.788. P < 0.0

## Discussion

The present study estimated GBM thickness in individuals subjected to renal biopsy at public hospitals in Salvador, Brazil, using both DM and OIM measurement techniques. A mean GBM value of 289 ± 44 nm was obtained using DM compared to 287 ± 48 nm using OIM. As expected, a larger number of estimates was performed in each glomerulus under OIM. Interestingly, similar coefficients of variation were obtained under both methods, which suggests that the observed differences in GBM values may not be attributable to discrepancies in the number of measurements taken. Furthermore, a strong degree of correlation was detected between the two methods used to estimate GBM. It is important to note that on average, the raw, uncorrected GBM values obtained using DM were 30% higher than those obtained using OIM; yet, this difference decreased to 10% following correction. It is important to highlight that correction is not typically applied when performing DM estimations, since this technique relies on the arbitrary perception of the observer. However, the data presented herein suggests that the application of correction using DM can result in greatly improved accuracy.

The GBM thickness values obtained in this study fell within the range proposed by other authors [3, 9, 12]. Some authors have reported increasing GBM thickness with age [10, 12]. While we did observe a trend indicating a small increase in GBM size in adults under both methods of measurement, this difference was not statistically significant. A possible explanation could be the limited sample size of the study, which resulted from restrictive inclusion and exclusion criteria, especially pertinent in older subjects. Unfortunately, it was not possible to obtain samples from patients aged between 41 and 60 years. Again, this may have resulted from strict inclusion criteria, as well as the occurrence of renal disease and comorbidities presented by patients subjected to renal biopsy within this age range. In this study excluded patients with any disease that could potentially affect the glomerular basement membrane thickness. For this reason, only patients with acute tubular necrosis or minimal change disease in proteinuric phase or in remission were included. Morita et al. (1988) did not find significative difference between estimates of GBM thickness made in kidneys of patients with MCD proteinuric phase of the disease or in remission [8].

In conclusion, DM represents an easy-to-use method for estimating GBM thickness, and is the method of choice employed by many pathologists in their daily routines. However, attention must be paid to the criteria used for estimating GBM thickness. According to Das et al. (1996), the shortest distance between the endothelial and podocyte membranes should be estimated. Furthermore, correction for non-perpendicular membrane sectioning must be applied to the mean of the obtained DM estimates.

## Supplementary Information

**Additional file 1.**

## Data Availability

The datasets used and/or analysed during the current study are available from the corresponding author request.
