# Supplementary material for: A comparative study of human glomerular basement membrane thickness using direct measurement and orthogonal intercept methods
Source: BMC Nephrol. 2022 Jan 10;23:23. doi: 10.1186/s12882-021-02634-1 (PMC8750857; doi:10.1186/s12882-021-02634-1)
Supplement: Supplementary file 1 — Additional file 1. [file 12882_2021_2634_MOESM1_ESM.docx]

Supplementar data.

**Figura1:** Frequency of thickness of glomerular basal membrane (GBM) for Sex and Age. Frequency of thickness of GBM for sex obtained by direct method - DM (A) and orthogonal intercept method – OIM (B). Frequency of thickness of GBM for age obtained by DM and OIM (C).
